# Salivary Chromogranin A (CgA) Response to the Noradrenaline Transporter Blocker Atomoxetine in Dogs

**DOI:** 10.3390/ani11102844

**Published:** 2021-09-29

**Authors:** Takanori Kooriyama, Abhijit Mukhopadhyay, George E. Moore, Niwako Ogata

**Affiliations:** 1 Department of Veterinary Science, School of Veterinary Medicine, Rakuno Gakuen University, Ebetsu 069-8501, Hokkaido, Japan; kooriyam@rakuno.ac.jp; 2 Department of Veterinary Clinical Sciences, College of Veterinary Medicine, Purdue University, West Lafayette, IN 47907, USA; mukhopad@purdue.edu; 3 Department of Veterinary Administration, College of Veterinary Medicine, Purdue University, West Lafayette, IN 47907, USA; gemoore@purdue.edu

**Keywords:** chromogranin A (CgA), cortisol, dog, hypothalamus–pituitary–adrenal (HPA), salivary stress marker, sympathetic adrenomedullar system (SAM)

## Abstract

**Simple Summary:**

Cortisol in peripheral samples (e.g., blood, saliva or hair) is commonly used for the assessment of stress in dogs. It primarily reflects the hypothalamus–pituitary–adrenal (HPA) axis responses and serves as a marker to indicate the rapid response via the sympathetic adrenomedullar system (SAM), which has not yet been well studied in dogs. This study aimed to evaluate chromogranin A (CgA), a known SAM activation marker, in saliva samples from laboratory dogs when the SAM response was pharmacologically induced. A selective noradrenaline transporter blocker, atomoxetine, was orally administered without causing any adverse responses in dogs to see if it increases salivary CgA. Three treatment groups were designed to determine whether CgA was increased only in the treatment with atomoxetine compared to a placebo or with pre-administration of dexmedetomidine followed by atomoxetine. Dexmedetomidine was included in the study because it is a selective alpha-2 adrenoreceptor agonist that inhibits central noradrenaline activity. The results were found to be consistent with our hypothesis and suggest that salivary CgA correlates with central noradrenaline activity, indicating that it can be a useful marker to assess SAM activity in dogs.

**Abstract:**

Since salivary chromogranin A (CgA) is one of the known sympathetic adrenomedullar system (SAM) stress markers in humans and pigs, this study aimed to investigate whether salivary CgA in dogs reflects SAM activation. Our hypothesis was that salivary CgA would increase when central noradrenaline was pharmacologically induced. A selective noradrenaline transporter blocker, atomoxetine, was orally administered without causing any aversive responses in nine laboratory dogs to see if it would increase salivary CgA. Three treatment groups (i.e., atomoxetine, placebo, and pre-administration of a selective alpha-2 adrenoreceptor agonist (dexmedetomidine) followed by atomoxetine) were prepared with a randomized crossover design. Saliva sample collection, heart rate measurement and behavior observation were performed at Time 0 (baseline) and at 30, 60, 90 and 150 min after each treatment administration. The results demonstrated that salivary CgA significantly increased at 90 min in the atomoxetine treatment (*p* < 0.05), whereas it was not observed in the other two treatments. The present study showed that salivary CgA was increased by atomoxetine-induced SAM activation. However, this increase was blocked if dexmedetomidine was pre-administered. Overall, the results indicate that salivary CgA is a potential candidate for SAM-mediated stress markers in dogs. Further study to determine the dynamics of salivary CgA will be helpful in its practical use.

## 1. Introduction

When an animal encounters stressors, there are two components to its response: the rapid response is facilitated by the sympathetic adrenomedullar system (SAM), which leads to the release of catecholamines from the adrenal medulla (e.g., noradrenaline and adrenaline), and sympathetic nerves are activated to prepare the body for fight or flight reactions. The other response is activation of the hypothalamus–pituitary–adrenal (HPA) axis, which results in an elevation of glucocorticoids. Dog stress levels can be evaluated by behavioral response, physiological response, hormones, and other analytes [1]. Each stress indicator has advantages or challenges in the given study design, and the use of multiple indicators, such as a combination of more than one indicator, is recommended [2,3,4]. Historically, in stress response studies of dogs, cortisol in peripheral samples, which primarily reflects HPA responses, has been commonly measured as a stress marker [5,6,7,8,9,10]. However, since stress responses are complicated and cannot be evaluated based on one component or one marker, the need to explore other markers, especially to reflect SAM responses, via noninvasive sample collection, is needed [1].

Chromogranin A (CgA) in peripheral samples is a known SAM activation marker in humans, dogs and pigs [11,12,13,14]. CgA is an acidic protein that is stored in the secretory granules of endocrine, neuroendocrine and neuronal cells [15,16]. It is a more stable analyte than catecholamines. Salivary CgA is produced and stored in the submandibular gland in humans [17], and in the salivary glands in pigs [12] and dogs [18]. Its secretion into saliva is induced by catecholamines. In studies with pigs [12,19], when pigs were immobilized for 1 min or 3 min with a nose snare, salivary CgA was detected as significantly higher than the baseline at 15 min post stress. At 30 min post stress, it was continuously higher than the baseline; however, it was not statistically significant. It was also reported that the longer immobilization with a nose snare (1 min vs. 10 min) caused longer elevation of salivary CgA after the stress [12,20]. Lensen et al., 2019 [3] used saliva samples in dogs to measure CgA and cortisol levels before and 20 min after the stressor (i.e., the strange situation test (SST)). The study showed that compared to the baseline, cortisol was increased in some dogs, while the CgA of all dogs decreased in the samples collected 20 min after the stressor. Thus, the authors speculated that CgA might reach its peak earlier than 20 min and then decrease at 20 min. Based on these results, it is assumed that CgA could be another valuable stress marker in dogs, especially for monitoring an acute stress response. However, it would be more helpful if the relationship between SAM activity and CgA is further examined under a controllable stressor to support this assumption.

In this study, we pharmacologically activated central noradrenaline by using atomoxetine, a selective noradrenaline transporter blocker. Atomoxetine binds to presynaptic noradrenaline transporters with minimal affinity for other monoamine transporters or receptors [21,22]. It is used to treat attention deficit hyperactivity disorder (ADHD) in children and adults. Although atomoxetine is not clinically used in veterinary medicine, Mattiuz et al., 2003 [23] studied beagle dogs and showed that the protein binding of atomoxetine is high (approximately 97%) and that its bioavailability via oral administration is high (approximately 74%).

Therefore, we expected that oral administration of atomoxetine would activate the SAM response in dogs, which is seen by an increase in salivary CgA. To confirm the SAM activity, we also measured salivary cortisol to see its change following central noradrenaline activity. Additionally, the salivary CgA and cortisol response to atomoxetine was compared with pre-administration of a dexmedetomidine oral transmucosal (OTM) gel (Sileo^®^; Zoetis Inc., Kalamazoo, MI, USA). Dexmedetomidine is a selective alpha-2 adrenoreceptor agonist, and Sileo^®^ is an FDA-licensed medication for dogs that inhibits central noradrenaline activity and its subsequent behavioral signs. Thus, we expected that the pre-administered dexmedetomidine gel would block CgA and the subsequent cortisol response to atomoxetine.

## 2. Materials and Methods

### 2.1. Animals

A total of nine laboratory beagles (3 females and 6 males) obtained from two universities were included in the study. All dogs were intact, and the mean ± SD of the age and body weight of the three females, which belonged to Purdue University, were 1.4 yrs and 7.6 ± 0.2 kg, while those of the six males, which belonged to Rakuno Gakuen University, were 7.1 ± 0.8 yrs and 13.5 ± 1.1 kg, respectively. All dogs were individually housed in each institutional animal laboratory, with visual and vocal contact with each other. They were considered healthy based on the results of a physical examination and bloodwork (complete blood count (CBC) and biochemical analysis) performed prior to the study. The dose of atomoxetine was determined based on the literature and a preliminary study conducted to evaluate the heart rate and behavioral responses as well as the salivary CgA concentrations with various doses. A study dose that did not cause severe adverse effects was chosen as determined by the physical examination and the bloodwork before and after the administration.

### 2.2. Study Protocol

The study was conducted with a randomized crossover design, and each dog was administered each of three treatments at the same time of the day. The dogs’ treatments were conducted during a window of time between 8:30 and 9:30 AM with an exact interval of 24 h between treatments: Treatment A (Tx A): atomoxetine (2.3–2.9 mg/kg doses), per os (PO); Treatment B (Tx B): lactose (2.3–2.9 mg/kg doses), PO as a placebo control treatment; and Treatment C (Tx C): the combination of pre-administration of a dexmedetomidine OTM gel (0.05–0.075 mg/kg doses), and atomoxetine (2.3–2.9 mg/kg doses), PO. Dexmedetomidine OTM gel was administered 30 min prior to atomoxetine administration at Time 0. The treatment order was determined by using an online randomization application (https://www.randomizer.org/, accessed on 2 March 2020). In the exam room, before the saliva samples were collected, the heart rate and behavioral changes due to possible adverse effects (e.g., tachycardia, hyperactivity) were monitored as a baseline (Time 0; immediately before the drug administration) and four time points per treatment (at 30, 60, 90 and 150 min after the administration). During the rest of the study time, the dogs were returned to a home cage, which was located next door to the exam room, with free access to water but not food, which was located next door to the exam room.

### 2.3. Saliva Sample Collection

Saliva samples were collected using SalivaBio Children’s Swab (Salimetrics, LLC, State College, PA, USA). Saliva flow was stimulated by presenting the smell of treats, and each saliva collection was performed within 2 min. The samples were frozen at −80 °C until assay.

### 2.4. CgA and Cortisol Assay

Salivary CgA and cortisol concentrations were measured using commercially available enzyme immunoassay (EIA) kits: CgA (Yanaihara Institute, Shizuoka, Japan) and cortisol (Salimetrics, State College, PA, USA), respectively. According to the literature, the kit in this study used antibodies directed against certain epitopes of CgA, which has a high cross-reactivity between humans and dogs [13]. Some clinical studies used the same kit to measure salivary CgA in dogs [3,18]. Nevertheless, salivary CgA is a relatively new marker in dogs, and a measurement protocol for CgA has not yet been established. Therefore, a preliminary study comparing saliva samples with no dilution and four-, eight-, and sixteen-fold dilutions was run, and the CgA concentrations were found to be consistent at different dilutions. We chose an eightfold dilution for this study protocol, as it was also used in the previous literature [3].

### 2.5. Statistical Analysis

Treatment effect differences in saliva samples (CgA and cortisol) were analyzed by two-way ANOVA, and the time and treatment effects were evaluated. When a significant difference was found, Tukey’s post hoc test was used to compare the baseline mean with the mean at a particular time point and to compare the mean values between treatments at each time point. All analyses were performed using SAS software (version 9.4, SAS Institute Inc, Cary, NC, USA) as two-tailed tests, and the level of significance was set at *p* < 0.05. Results in the figures are shown as the mean ± SEM.

## 3. Results

### 3.1. Salivary CgA

**Figure 1:**
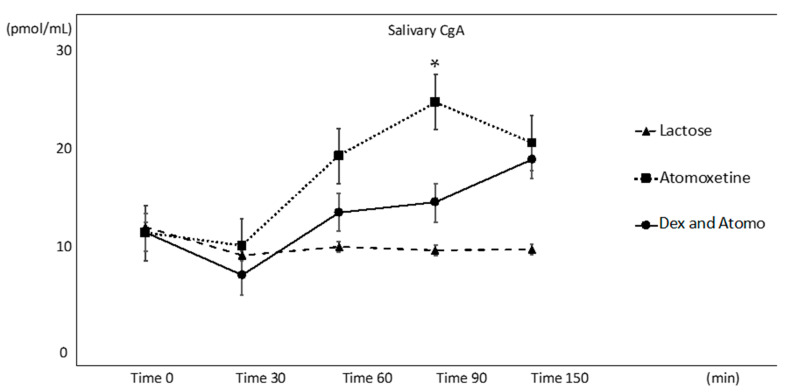
Salivary CgA response to three treatments. Error bars reflect SEM. Asterisk indicate. *p* < 0.05.

The salivary CgA profiles of the three treatments (Tx A, B, and C) and changes over time are shown in Figure 1. After 30 min (Time 30), the CgA concentration was found to be greater in both Tx A (atomoxetine) and Tx C (dexmedetomidine and atomoxetine) than in the placebo Tx B (lactose). Importantly, the CgA concentrations at 60 and 90 min were found to be much higher in Tx A than the CgA concentrations found in Tx C at the corresponding time points (Figure 1), although the difference was not statistically significant. In Tx A, the CgA concentration at Time 90 was significantly higher than the CgA concentration at Time 30 (*t* = −3.75; *p* = 0.021). Time 90 in Tx A was significantly higher than that in Tx B (*p* = 0.014) but not that in Tx C (*p* = 0.3614).

### 3.2. Salivary Cortisol

**Figure 2:**
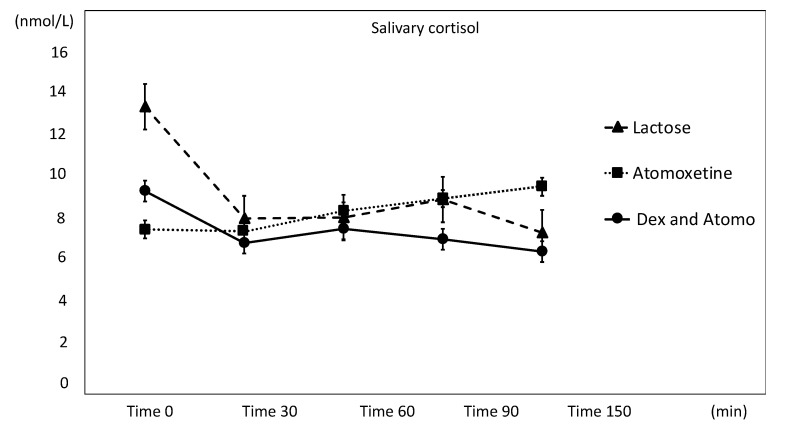
Salivary cortisol response to three treatments. Error bars reflect SEM.

The salivary cortisol results of the three treatments (Tx A, B, and C) are shown in Figure 2. The cortisol concentrations were not significantly different from baseline to any time point in any of the three treatments (F = 1.15; *p* = 0.338).

### 3.3. Heart Rate and Behavior Responses

Controlling for sex/location, the heart rate did not significantly differ over time (F = 1.56; *p* = 0.190), nor was there an interaction between time and treatment group (F = 0.42; *p* = 0.907). Compared to the baseline (time 0), the overall heart rate per minute was changed in a range of −32 to 30% in Tx A, −33 to 22% in Tx B, and −50 to 0% in Tx C. All observed heart rates were in the clinically healthy heart rate range. Additionally, no aversive responses in behaviors were observed at each time point in any of the three treatments.

## 4. Discussion

The main finding of this study was that salivary CgA increased primarily with pharmacologically induced sympathetic activation by orally administering the noradrenaline transporter blocker atomoxetine (Tx A) and to a much lesser extent in the presence of the antagonist dexmedetomidine (Tx C). Although it was not statistically significant, the increase in CgA in Tx C after pre-administration of dexmedetomidine (before atomoxetine administration) was lower than that in Tx A (atomoxetine only). Salivary CgA has recently attracted attention as a possible marker for measuring SAM activity in pigs and dogs [3,12,19]; however, the published literature that supports this underlying idea is limited, especially in dogs. There are several advantages to using CgA in dogs. Since it can be measured in saliva samples, it does not require the specific skill set for sample collection compared to blood collection. Generally speaking, saliva collection is less invasive, less costly, and is safer to collect, making it more applicable for clinical studies, especially when study animals do not live in a laboratory setting. However, there are not many saliva markers that are explored in dogs for evaluating a stress response. Cortisol is one of the stress markers that is most commonly used; however, it primarily reflects the HPA axis. Since the HPA axis responds slower than the autonomic nervous system (ANS), if only a cortisol level is used, a study design needs to be carefully interpreted as the intensity of the stressor and the timing of sample collection affect the outcome [24]. It is important to keep in mind that cortisol alone does not sufficiently reflect the complexity of all stress responses [25,26] and the use of multiple markers of various stress responses can cover all responses in animals [27].

In this study, we pharmacologically induced norepinephrine responses and the study protocol was strictly planned to be consistent, including the time of the treatment administration in each condition. It is known that for CgA in humans, saliva levels demonstrate a circadian rhythm in the saliva, in which CgA levels in saliva reach a peak upon waking, decrease within 1 h, and then remain at a low level throughout the day [28]. In dogs, however, when salivary levels of CgA levels in the saliva were measured every 4 h, no circadian variation was detected [18], which can be another advantage for using this marker in dogs.

To our knowledge, this is the first study to show the correlation between salivary CgA and central noradrenaline activity in dogs.

According to a previous study, the time of maximal plasma concentration (T_max_) of orally administered (2 mg/kg) atomoxetine is 1.3 ± 0.3 h (Mean ± SEM) [23]. The time course of the salivary CgA in our study corresponded to the above value. We compared the CgA response after atomoxetine administration (Tx A) with placebo (Tx B) as well as the pre-administration of dexmedetomidine OTM gel (Tx C). According to the Sileo^®^ package insert [29], T_max_ is approximately 0.6 h, and the drug can be redosed with at least a two-hour interval between dosages up to five doses during one stressful (e.g., noise) event. Thus, when atomoxetine was administered in Tx C, it was considered that the pre-administration of dexmedetomidine OTM gel was effective and could inhibit the release of noradrenaline from noradrenergic neurons. Accordingly, the CgA concentrations in Tx C were lower than the CgA concentrations in Tx A from Times 30 to 150. This result also supports the study hypothesis that salivary CgA reflects central noradrenaline activity, which was therefore blocked by dexmedetomidine OTM gel in Tx C.

On the other hand, salivary cortisol gradually increased after atomoxetine administration in Tx A. However, this cortisol level did not significantly differ among the three treatments. This nonsignificant result might be related to the dose of atomoxetine used in this study. In human studies where atomoxetine was used to activate central noradrenaline, a dose of 40 mg [30] or 60 mg [31] per adult was used. However, we chose this study dose with caution based on the Animal Poison Control Center (APCC; American Society for the Prevention of Cruelty to Animals (ASPCA), New York City, NY, USA) toxicology database report, where Stern and Schell reported that 2.2 mg/kg oral administration in dogs could cause stimulatory signs such as hyperactivity or tachycardia [32]. Thus, after the preliminary study, we chose a dose range of 2.3–2.9 mg/kg, which did not cause any adverse effects observed through heart rate monitoring and behavior observation. To determine the sufficient dose of atomoxetine that can trigger the HPA axis response in dogs, separate studies are needed.

The present study had some limitations. We used laboratory beagle dogs, which may not have adequately represented the population of pet dogs. The study did not include other variables that reflect noradrenaline responses (e.g., blood pressure and blood glucose concentrations). In addition, the study design was not blinded, which may at least have impacted the judgment of behavior. Despite these limitations, the saliva sample analysis of CgA supported our hypothesis, and this study suggested that salivary CgA can be a useful SAM activity marker in dogs. The findings of this study should lead to further studies such as evaluating the duration of salivary CgA elevation after stressful stimuli. Considering the multiple reports indicating that the prevalence of fear- and anxiety-related problems in dogs is 20−50% [33,34,35,36], addressing their welfare based on an accurate evaluation of stress levels is needed. Overall, future investigation of salivary CgA dynamics as a stress response will enable us to determine the sensitivity and accuracy of CgA as a suitable marker.

## 5. Conclusions

In the present study, the results indicated that salivary CgA reflects central noradrenaline activity in dogs. Although further studies using common stressors for pet dogs, such as loud noises, are needed to validate the clinical application, salivary CgA could be used as a potential marker of acute stress in dogs.

## Data Availability

The data presented in this study are available on request from the corresponding author.

## References

[1] Chmelíková E., Bolechová P., Chaloupková H., Svobodová I., Jovičić M., Sedmíková M. (2020). Salivary cortisol as a marker of acute stress in dogs: A review. Domest. Anim. Endocrinol..

[2] Hekman J.P., Karas A.Z., Dreschel N.A. (2012). Salivary cortisol concentrations and behavior in a population of healthy dogs hospitalized for elective procedures. Appl. Anim. Behav. Sci..

[3] Lensen R.C.M.M., Moons C.P.H., Diederich C. (2019). Physiological stress reactivity and recovery related to behavioral traits in dogs (*Canis familiaris*). PLoS ONE.

[4] Polgár Z., Blackwell E.J., Rooney N.J. (2019). Assessing the welfare of kennelled dogs—A review of animal-based measures. Appl. Anim. Behav. Sci..

[5] Accorsi P.A., Carloni E., Valsecchi P., Viggiani R., Gamberoni M., Tamanini C., Seren E. (2008). Cortisol determination in hair and faeces from domestic cats and dogs. Gen. Comp. Endocrinol..

[6] Beerda B., Schilder M.B., Janssen N.S., Mol J.A. (1996). The use of saliva cortisol, urinary cortisol, and catecholamine measurements for a noninvasive assessment of stress responses in dogs. Horm. Behav..

[7] Dreschel N.A., Granger D.A. (2009). Methods of collection for salivary cortisol measurement in dogs. Horm. Behav..

[8] Palme R. (2019). Non-invasive measurement of glucocorticoids: Advances and problems. Physiol. Behav..

[9] Rooney N.J., Gaines S.A., Bradshaw J.W.S. (2007). Behavioural and glucocorticoid responses of dogs (Canis familiaris) to kennelling: Investigating mitigation of stress by prior habituation. Physiol. Behav..

[10] Steiss J.E., Schaffer C., Ahmad H.A., Voith V.L. (2007). Evaluation of plasma cortisol levels and behavior in dogs wearing bark control collars. Appl. Anim. Behav. Sci..

[11] Akiyoshi H., Aoki M., Kumagai D., Saleh N., Noda K., Shimada T., Sugii S., Ohashi F. (2005). Emzyme-linked immunosorbent assay for detection of canine chromogranin A by use of immunological cross-reactivity of rabbit anti-bovine chromogranin A antibody. J. Vet. Med Sci..

[12] Escribano D., Soler L., Gutiérrez A.M., Martínez-Subiela S., Cerón J.J. (2013). Measurement of chromogranin A in porcine saliva: Validation of a time-resolved immunofluorometric assay and evaluation of its application as a marker of acute stress. Animal.

[13] Stridsberg M., Pettersson A., Hagman R., Westin C., Höglund O. (2014). Chromogranins can be measured in samples from cats and dogs. BMC Res. Notes.

[14] Taupenot L., Harper K.L., O’Connor D.T. (2003). The Chromogranin–Secretogranin Family. N. Engl. J. Med..

[15] Banks P., Helle K. (1965). The release of protein from the stimulated adrenal medulla. Biochem. J..

[16] Helle K.B. (1966). Some chemical and physical properties of the soluble protein fraction of bovine adrenal chromaffin granules. Mol. Pharmacol..

[17] Saruta J., Tsukinoki K., Sasaguri K., Ishii H., Yasuda M., Osamura Y.R., Watanabe Y., Sato S. (2005). Expression and localization of chromogranin A gene and protein in human submandibular gland. Cells Tissues Organs.

[18] Kanai K., Hino M., Hori Y., Nakao R., Hoshi F., Itoh N., Higuchi S. (2008). Circadian variations in salivary chromogranin a concentrations during a 24-hour period in dogs. J. Vet. Sci..

[19] Escribano D., Fuentes-Rubio M., Cerón J.J. (2014). Salivary testosterone measurements in growing pigs: Validation of an automated chemiluminescent immunoassay and its possible use as an acute stress marker. Res. Vet. Sci..

[20] Huang Y., Liu Z., Liu W., Yin C., Ci L., Zhao R., Yang X. (2017). Short communication: Salivary haptoglobin and chromogranin A as non-invasive markers during restraint stress in pigs. Res. Vet. Sci..

[21] Gehlert D.R., Gackenheimer S.L., Robertson D.W. (1993). Localization of rat brain binding sites for [3H] tomoxetine, an enantiomerically pure ligand for norepinephrine reuptake sites. Neurosci. Lett..

[22] Wong D.T., Threlkeld P.G., Best K.L., Bymaster F.P. (1982). A new inhibitor of norepinephrine uptake devoid of affinity for receptors in rat brain. J. Pharmacol. Exp. Ther..

[23] Mattiuz E.L., Ponsler G.D., Barbuch R.J., Wood P.G., Mullen J.H., Shugert R.L., Li Q., Wheeler W.J., Kuo F., Conrad P.C. (2003). Disposition and metabolic fate of atomoxetine hydrochloride: Pharmacokinetics, metabolism, and excretion in the Fischer 344 rat and beagle dog. Drug Metab. Dispos..

[24] Cobb M.L., Iskandarani K., Chinchilli V.M., Dreschel N.A. (2016). A systematic review and meta-analysis of salivary cortisol measurement in domestic canines. Domest. Anim. Endocrinol..

[25] Csoltova E., Martineau M., Boissy A., Gilbert C. (2017). Behavioral and physiological reactions in dogs to a veterinary examination: Owner-dog interactions improve canine well-being. Physiol. Behav..

[26] Stollar O., Moore G., Mukhopadhyay A., Gwin W., Ogata N. (2021). Effects of a single dose of oral gabapentin in dogs during a veterinary visit: A double-blind, placebo-controlled study. J. Am. Vet. Med Assoc..

[27] Hekman J.P., Karas A.Z., Sharp C.R., Grafton N. (2014). Psychogenic stress in hospitalized dogs: Cross species comparisons, implications for health care, and the challenges of evaluation. Animals.

[28] Den R., Toda M., Nagasawa S., Kitamura K., Morimoto K. (2007). Circadian rhythm of human salivary chromogranin A. Biomed. Res..

[29] ZoetisUS.com. https://www.zoetisus.com/contact/pages/product_information/msds_pi/pi/sileo.pdf.

[30] Warren C.M., van den Brink R.L., Nieuwenhuis S., Bosch J.A. (2017). Norepinephrine transporter blocker atomoxetine increases salivary alpha amylase. Psychoneuroendocrinology.

[31] Chamberlain S.R., Müller U., Cleary S., Robbins T.W., Sahakian B.J. (2007). Atomoxetine increases salivary cortisol in healthy volunteers. J. Psychopharmacol..

[32] Stern L., Schell M. (2018). Management of Attention-Deficit Disorder and Attention-Deficit/Hyperactivity Disorder Drug Intoxication in Dogs and Cats: An Update. Vet. Clin. North Am.-Small Anim. Pract..

[33] Tiira K., Sulkama S., Lohi H. (2016). Prevalence, comorbidity, and behavioral variation in canine anxiety. J. Vet. Behav..

[34] Kurachi T., Irimajiri M., Mizuta Y., Satoh T. (2017). Dogs predisposed to anxiety disorders and related factors in Japan. Appl. Anim. Behav. Sci..

[35] Dinwoodie I.R., Dwyer B., Zottola V., Gleason D., Dodman N.H. (2019). Demographics and comorbidity of behavior problems in dogs. J. Vet. Behav..

[36] Salonen M., Sulkama S., Mikkola S., Puurunen J., Hakanen E., Tiira K., Araujo C., Lohi H. (2020). Prevalence, comorbidity, and breed differences in canine anxiety in 13,700 Finnish pet dogs. Sci. Rep..

